# Extracurricular physical exercise and self-education expectations among Chinese teenagers

**DOI:** 10.3389/fpsyg.2025.1518100

**Published:** 2025-02-12

**Authors:** Qin Xiao, Fengyun Tang

**Affiliations:** ^1^School of Physical Education, Hunan First Normal University, Changsha, Hunan, China; ^2^School of Education, Zhanjiang University of Science and Technology, Zhanjiang, Guangdong, China

**Keywords:** extracurricular physical exercise, group differences, ordered logistic regression model, self-education expectation, teenagers

## Abstract

Drawing on data from 16,540 seventh and ninth graders from the China Education Survey (CES), the study employs descriptive statistics and Ordinal Logistic Regression (Ologit) models. These methods dissect the variances in self-education expectations among different youth groups and unravel the effects and heterogeneity of after-school physical exercises on these expectations. The study has two main findings: First, there is a marked difference in self-education expectations between adolescents who engage in extracurricular physical exercise and those who do not. Specifically, the group participating in these activities shows a 20.62% higher expectation than their non-participating peers, male students exhibit a 26.57% increase in self-education expectations, while female students show a 15.21% increase. Then, the impact of extracurricular physical exercise on self-education expectations is significantly influenced by cognitive abilities, academic performance, health status, confidence level, and family factors. The most pronounced effects are observed in self-confidence (*b* = 0.6490, *p* < 0.01), cognitive ability (*b* = 0.2363, *p* < 0.01), and health status (*b* = 0.1541, *p* < 0.01). The findings suggest that interventions to increase physical exercise among adolescents should be sensitive to the diverse needs of different demographic groups and consider the key role of familial background and socio-economic conditions.

## Background

The escalating decline in physical fitness among youth globally, particularly in China, has raised concerns about the healthy development of young people (Zhu et al., 2019; Zhu et al., 2023; Chen et al., 2020). This trend is notably pronounced among Chinese students, who grapple with an intense academic burden due to a relative scarcity of high-quality educational resources. Historically, extracurricular physical exercise has been undervalued in this context, often perceived as a leisurely distraction detracting from core academic pursuits (Zubing et al., 2021). This perception has contributed to a widespread neglect of physical activity, adversely impacting students’ physical health. In response, initiatives like the Healthy China Action (2019–2030) have been introduced by the Chinese government and relevant bodies, aiming to enhance youth physical fitness and promote a harmonious balance between academic learning and physical exercise (National Health and Wellness Commission of the People’s Republic of China, n.d.; General Administration of Sports Ministry of Education, n.d.; General Office of the Central Committee of the Communist Party, n.d.).

In a significant policy shift, the “double reduction” policy was introduced in July 2021 (Yang and Yuhang, 2022), marking a transition from a high-pressure, performance-centric education model to a more holistic, student-centered approach to quality education. Despite this shift, the entrenched “performance-based” orientation of the education system and pervasive ‘education anxiety’ among families continue to exert pressure, influencing parental expectations and shaping adolescents’ own educational aspirations. Self education expectation refers to an individual’s expectation of the educational level and degree they hope to achieve in the future based on their past learning and life experiences and current learning situation. It is a realistic evaluation of their educational goals (Abu-Hilal, 2000; Li et al., 2020). These aspirations, or self-education expectations, encompass adolescents’ psychological ambitions and willingness to pursue certain educational levels or complete specific educational stages (Yao et al., 2020). Serving as a motivational force, these expectations are instrumental in driving learning motivation, focus, persistence, and self-discipline, ultimately impacting educational attainment and career success (Rothon et al., 2011; Zhu et al., 2019).

Recent scholarly attention has increasingly affirmed the positive impact of physical exercise on students’ academic performance (Coe et al., 2013; Tomporowski et al., 2011; Giusti et al., 2024; Alvarez-Bueno et al., 2017). The interplay between physical activity and academic achievement has thus garnered substantial academic interest. Studies indicate that physical exercise not only enhances adolescents’ physical fitness through health interventions but also influences their self-education expectations through a myriad of factors, including physiological, neurological, cognitive regulation, and social psychology (Gong et al., 2020). At the physiological and neurological levels, research suggests that exercise can promote brain development, enhance brain plasticity and activation energy, and subsequently improve cognitive processes such as perception, memory, and thinking, all of which potentially boost adolescents’ academic abilities and self-education expectations (Hu and Lv, 2018; Loprinzi et al., 2019; Szuhany et al., 2015). Furthermore, cognitive studies have shown that sports enhance adolescents’ executive function, impacting lifelong academic achievement (Alloway and Alloway, 2010; Voss et al., 2013; Borkertiene et al., 2019). The “executive function hypothesis” posits that physical activity fosters cognitive ability and executive function, crucial for academic performance and educational goal orientation (Ballester et al., 2018; Soltani Kouhbanani et al., 2020).

Psychosocially, the self-efficacy theory elucidates the mechanism through which physical activity affects self-education expectations (Bandura, 2004; Mesquita et al., 2012). It emphasizes the strong correlation between physical activity and self-efficacy, a core determinant of behavioral motivation (Zhaohui, 2020). Physical activity, by rebuilding positivity and self-concept, particularly in individuals frequently facing setbacks, exerts a significant positive influence on self-efficacy (Pu et al., 2017; Wei, 2018; Guo and Wei, 2020). Studies have found that physical exercise positively affects self-concept and self-efficacy (Lian-Cheng et al., 2015; Zhaohui, 2020), improving physical and mental health, psychological quality, frustration tolerance, and interpersonal skills (Higgins et al., 2014). This, in turn, enhances self-esteem, self-confidence, and positive personality traits, thereby fostering higher self-education expectations (Gong et al., 2020; Moratal et al., 2020). Educational psychology research further indicates that sports experiences can amplify cognitive, self-improvement, and accessory drives in adolescents, promoting effective learning motivation and higher goal pursuit (Phan, 2014; Lei, 2019; Higgins et al., 2014).

Lastly, it is acknowledged that the impact of physical exercise on self-education expectations and academic achievement varies with individual characteristics like gender, age, personality, cognitive and non-cognitive abilities, and family background (Watson et al., 2017). While existing literature predominantly focuses on the relationship between in-class sports and academic achievement (Domina et al., 2011; Watson et al., 2017), there is a dearth of normative empirical studies examining the actual relationship between physical exercise and self-education expectations, especially in the context of China’s “double reduction” policy.

Hence, the present study aims to address these knowledge voids by leveraging micro-survey data encompassing 16,540 Chinese adolescents aged between 12 and 18 years, participating in the China Education Panel Survey (CEPS). The primary objective is to assess the disparities in self-education aspirations among diverse adolescent subgroups, characterized by their distinct attributes. A specific focus will be on examining whether extracurricular physical exercise, on an aggregate level, enhances the self-education aspirations of adolescents. Furthermore, this study aims to delve into the gender-specific differences, particularly comparing male and female adolescents in this context. Ultimately, the objective is to unpack the underlying mechanism through which extracurricular physical exercise influences the self-education aspirations of adolescents.

## Data and methods

### Data

This study uses data from the China Education Panel Survey (CEPS) 2013–2014 baseline survey designed and implemented by the China Survey and Data Center of Renmin University of China. The CEPS is a nationally representative large-scale education tracking survey that aims to reveal the impact of family, school, community, and macro-social structures on individual educational outcomes and to investigate the process by which educational outcomes occur over the life course of individuals. This study uses data from a baseline survey from 2013 to 2014 with two contemporaneous cohorts of first grade (Year 7) and third grade (Year 9) as the starting point of the survey, with a sample of approximately 20,000 students in 112 schools and 438 classes in 28 counties (counties, districts, and cities). After matching data, samples with missing primary and abnormal variables were excluded, and 16,540 valid samples were obtained.

### Variables

#### Dependent variable

The dependent variable is the adolescent self-education expectation, which corresponds to the question of CEPS, “How far do you want to study? “The dependent variable is the self-education expectation of adolescents, which is set as 1 or 2 for students who answered “not to study now,”2 for junior high school graduates, 3 for junior college/technical school, and 4, 5, 6, 7, 8, 9 for vocational high school, general high school, university college, university bachelor’s degree, master’s degree, and doctoral degree, respectively to form an ordinal variable with values from 1 to 9.

### Independent variable

The independent variable was extracurricular physical activity. Extracurricular sports activities, as a form of healthy practice, are organized and unorganized sports activities conducted outside of school curriculum, and are part of students’ self-directed physical activities (Cleland et al., 2005; Fernandez-Lazaro and Fernández-Lázaro, 2023). The corresponding question in the CEPS questionnaire was “On average, what was your daily extracurricular activity schedule last weekend? homework/attend extracurricular classes/participate in extracurricular sports/read books/watch TV and many others.” If the student answered “participate in extracurricular sports” and gave the specific length of sports, then the value is set to 1.

### Control variables

To control the influence of other factors, a series of control variables were selected, including students’, parental and family characteristics, and school characteristics. The main variables and their descriptive statistics are shown in Table 1.

**Table 1:** Variable definitions and descriptive statistics.

Type	Variables	Definition and settings	Average value	Standard deviation	Minimum value	Maximum value
Dependent variables	Self-education expectations	Adolescent self-education expectations: 7 years = 1, 9 years = 2, junior high school graduation = 3, …… masters = 8, PhD = 9	5.7332	1.6604	1	9
Independent variables	Extracurricular physical exercise	Whether to participate in physical exercise during the weekend, yes = 1, no = 0	0.6086	0.4880	0	1
Control variables	student Gender	Male = 1, Female = 0	0.5113	0.4998	0	1
	Cognitive ability	Standardized scores on student cognitive ability tests	0.0117	0.8592	−2.0289	2.7099
	academic Performance	scores in language, math, and English	69.9685	8.8073	9.0517	97.9767
	Health status	Student self-assessment of health: very good/better set to 1, fair/not so good/very bad set to 0	0.7279	0.4450	0	1
	Whether or not to live on campus	Whether the student usually lives at school, living at school = 1, not living at school = 0	0.3217	0.4671	0	1
	Self-confidence	Do you have confidence in your future: no confidence at all/not very confident = 0, more confident/very confident = 1	0.8541	0.3529	0	1
	Persistence	Even if the homework takes a long time to finish, I still keep trying my best to do it: disagree/not quite agree = 0, more agree/ agree = 1	0.8607	0.3462	0	1
	Account type	Agricultural households = 1, non-agricultural households = 0	0.5599	0.4964	0	1
	Parenting	Maximum number of years of Parental education	10.7080	3.0668	1	19
	Family economic conditions	Poor families = 1, non-poor families = 0	0.1178	0.3225	0	1
	Only one child or not	Only one child = 1, non-only one child = 0	0.4373	0.4960	0	1
	Whether the parents live at home	Whether parents live together at home, one parent at home or neither parent at home = 1; both parents at home = 0.	0.2305	0.4212	0	1
	Mobility	In-province or inter-province mobile students = 1, local non-mobile students = 0	0.1732	0.3784	0	1
School characteristics	Nature of school	Nature of school, public school = 1, private school = 0	0.9241	0.2648	0	1
	School ranking	School ranking in the county (district), medium and below = 1, medium and above = 2, best = 3	2.0359	0.6449	1	3
	Location type	The location of the school is a rural township = 1; marginal urban area and urban–rural area = 2, central urban area of the city/county = 3	2.0201	0.8648	1	3
Regional characteristics	Regional distribution	East = 1, Central = 2, West = 3	1.6882	0.8309	1	3

## Methods

Utilizing descriptive and inferential statistical techniques, this study delves into the self-education aspirations of adolescents across various characteristics and compares the disparities among these subgroups. Subsequently, an ordered logistic regression model is employed, with extracurricular physical exercise serving as the independent variable and self-education aspirations as the dependent variable. This approach allows for a comprehensive examination of the impact and heterogeneity of extracurricular physical exercise on the self-education aspirations of distinct adolescent groups, while also highlighting any group-specific differences in the influence of physical exercise. Gender, a significant factor in this study, is accounted for through separate analyses conducted for male and female adolescents. Furthermore, to gain a deeper understanding, mechanism analysis methods are employed to unpack the underlying mechanisms through which extracurricular physical exercise influences adolescents’ self-education aspirations, specifying the precise pathways and channels of this influence.

In this study, the dependent variable is a multi-categorical ordered variable with typical discrete nature, so the ordered logistic regression model is used for analysis. Let the dependent variable have a total *k* rank (*k* = 9 in this study). Let the cumulative probability that the rank j(j=1,2,⋯,k) of the educational expectation rank category $p\left(y=j|x\right)$ is greater than or equal to j be:


(1) $$p\left(y\geq j|x\right)=p\left(y=j|x\right)+L+p\left(y=k|x\right)$$

Then the ordered logit regression is defined as:


(2) $$\begin{array}{l} \text{Logit}P_{j}=\text{Logit}\left[P\left(y\geq j|x\right)\right]\\ =\ln\frac{P\left(y\geq j|x\right)}{1-P\left(y\geq j|x\right)}=-\alpha_{j}+\sum_{j=1}^{n}\beta_{j}x_{i} \end{array}$$

$\alpha_{j}$ is the intercept of the model; $\beta_{i}$ is the coefficient corresponding to the $x_{i}$ independent variable. After obtaining the parameter estimates, the cumulative probability is expressed as:


(3) $$p\left(y\geq j|x\right)=\frac{1}{1-\exp\left(-\alpha_{j}+\sum_{j=1}^{n}\beta_{j}x_{i}\right)}$$

Although the size of the ordered logistic regression model’s regression coefficient represents a relative proportion, it cannot be directly used to measure the impact of the independent variable on the dependent variable. Therefore, combining the direction of the regression coefficient and the corresponding OR value (Odds Ratio, OR) of the regression coefficient explains the model. The following equation converts the ordered logistic regression model regression coefficient into an OR value:


(4) $$\text{OR}=\exp\left[\beta_{j}\left(b-a\right)\right]$$

This study employed extracurricular physical exercise as the independent variable and self-education expectations as the dependent variable to construct six statistical models. These models aimed to investigate the influence of extracurricular physical exercise on the self-education aspirations of male and female adolescent subgroups characterized by various attributes, as well as to explore potential group differences. All data processing and statistical analyses were conducted using STATA 17.0 software. For univariate analysis, this study relied on the *T*-test and *F*-test. Statistical significance was determined at the 5% level.

## Results

### Differences in self-education expectations among adolescent groups with different characteristics

Table 2 lists the average levels of self-education expectations among adolescent groups with different characteristics. Table 2 reveals that female adolescents (5.899) have higher self-education expectations than male adolescents (5.575). Female and male adolescents who participate in extracurricular physical exercise have higher self-education expectations (6.045, 5.765) than female and male adolescents who do not participate in extracurricular physical exercise (5.703, 5.227), respectively. The 12–13-year-old female adolescent group (6.236) has higher self-education expectations than the 14–18-year-old female group (5.757), and males follow similarly. Han female adolescents (5.905) have slightly higher self-education expectations than minority female adolescents (5.830), and Han male adolescents (5.579) have slightly higher self-education expectations than minority female adolescents (5.533). The self-education expectations of rural female adolescents (5.375) are much lower than those of urban female adolescents (6.143), while the gap between self-education expectations of rural male adolescents (5.701) and urban male adolescents (5.836) is much smaller than that of females, indicating a little difference in self-education expectations between urban and rural male adolescents. Whether male or female, their self-education expectations increase as their cognitive ability or academic performance improves. The self-education expectations of female and male adolescents who lack confidence (5.134, 4.613) are far lower than those of more confident female and male adolescents (6.036, 5.739). Healthy female adolescents (5.992) have higher self-education expectations than unhealthy female adolescents (5.674), while healthy male adolescents (5.677) have higher self-education expectations than unhealthy male adolescents (5.269). Regarding family characteristics, whether male or female, the higher the education level of their parents, the higher the individual’s self-education expectations, the better the family’s economic conditions, and the higher the individual’s self-education expectations.

**Table 2:** The average self-education expectation value of different characteristic youth groups.

		Expectation of self-education			*p*-Value
		Female (X ± S)	Male (X ± S)	All students (X ± S)	
Extracurricular physical exercise	Participation	6.045 ± 1.446	5.765 ± 1.673	5.894 ± 1.579	<0.001^#^
	Not involved	5.703 ± 1.615	5.227 ± 1.863	5.691 ± 1.669	
Gender	Female			5.899 ± 1.530	<0.001^#^
	Male			5.575 ± 1.762	
Age	12–13 years old	6.236 ± 1.430	5.937 ± 1.706	6.092 ± 1.576	<0.001^#^
	14–18 years old	5.757 ± 1.548	5.445 ± 1.763	5.594 ± 1.671	
Nation	the Han nationality	5.905 ± 1.525	5.579 ± 1.760	5.738 ± 1.657	<0.001^#^
	Minority nationality	5.830 ± 1.583	5.533 ± 1.787	5.681 ± 1.694	
Domicile	Rural area	5.375 ± 1.787	5.701 ± 1.575	5.532 ± 1.696	<0.001^#^
	Town	6.143 ± 1.435	5.836 ± 1.692	5.988 ± 1.577	
Cognitive ability	Low	5.306 ± 1.653	4.910 ± 1.865	5.099 ± 1.778	<0.001^+^
	Medium	5.962 ± 1.464	5.636 ± 1.683	5.798 ± 1.586	
	High	6.383 ± 1.291	6.164 ± 1.517	6.271 ± 1.415	
Academic performance	Poor	4.868 ± 1.727	4.764 ± 1.845	4.797 ± 1.808	<0.001^+^
	Medium	5.803 ± 1.432	5.745 ± 1.592	5.773 ± 1.516	
	Good	6.434 ± 1.267	6.393 ± 1.424	6.418 ± 1.330	
Self-confidence	Lack of confidence	5.134 ± 1.713	4.613 ± 1.861	4.873 ± 1.807	<0.001^#^
	Relatively confident	6.036 ± 1.452	5.739 ± 1.689	5.883 ± 1.585	
Health condition	Unhealthy	5.674 ± 1.623	5.269 ± 1.865	5.483 ± 1.752	<0.001^#^
	Health	5.992 ± 1.480	5.677 ± 1.714	5.827 ± 1.615	
Parental education level	≤ 9 years	5.622 ± 1.601	5.249 ± 1.795	5.431 ± 1.713	<0.001^+^
	9 < & < 15	5.992 ± 1.426	5.669 ± 1.678	5.824 ± 1.570	
	≥ 15 years	6.556 ± 1.220	6.409 ± 1.477	6.483 ± 1.355	
Family economic conditions	Poor families	5.712 ± 1.702	5.397 ± 1.879	5.548 ± 1.803	<0.001^+^
	Average household	5.863 ± 1.514	5.527 ± 1.750	5.694 ± 1.645	
	Affluent families	6.380 ± 1.346	6.025 ± 1.642	6.179 ± 1.530	

^#^Independent samples *t*-test was used, ^+^*F*-test was used. X and S denote the sample mean and standard deviation, respectively.

### The impact of extracurricular physical exercise on the self-education expectations of adolescents with different characteristics

This study used extracurricular physical exercise as the independent variable and self-education expectations as the dependent variable to establish six statistical models to analyze the impact of extracurricular physical exercise on self-education expectations of different characteristics of male and female adolescent groups and their group differences (Table 3). Models 1–3 reported the regression results of the full, female, and male samples with only the addition of independent variables and no control variables. Models 4–6 reported the regression results of the full, female, and male samples with the addition of independent and all control variables. According to Models 1 and 4, we discovered that the estimated coefficients of extracurricular physical exercise on adolescent self-education expectations were 0.4258 and 0.1875, respectively, and both passed the significance level test of 1% (* * *). This indicates that extracurricular physical exercise has a positive intervention effect on teenagers’ self-education expectations, which is conducive to significantly improving teenagers’ self-education expectations. The Pseudo R2 value of Model 4 is greater than that of Model 1, presenting that the explanatory power of Model 4 is relatively stronger than that of Model 1. Then, we calculated the OR value corresponding to the independent variable coefficient in Model 4 based on Equation 3 in introducing statistical methods mentioned earlier, which is 1.2062. This indicates that the likelihood of self-education expectations of the group of teenagers participating in extracurricular physical exercise increasing by one or more levels will increase by an average of 20.62% compared to the group of teenagers who do not participate in extracurricular physical exercise. Therefore, extracurricular physical exercise is an effective means to enhance teenagers’ self-education expectations.

**Table 3:** The ordered logistic regression model baseline regression estimates.

Dependent variable Self education expectations	Model 1	Model 2	Model 3	Model 4	Model 5	Model 6
	Total sample	Female	Male	Total sample	Female	Male
	Coefficient (SE)	Coefficient (SE)	Coefficient (SE)	Coefficient (SE)	Coefficient (SE)	Coefficient (SE)
Extracurricular physical exercise	0.4258^***^	0.3835^***^	0.5345^***^	0.1875^***^	0.1416^***^	0.2357^***^
	(0.0273)	(0.0385)	(0.0395)	(0.0274)	(0.0387)	(0.0396)
Gender				−0.0940^***^		
				(0.0269)		
Age				−0.1297^***^	−0.1389^***^	−0.1266^***^
				(0.0108)	(0.0161)	(0.0147)
Domicile				−0.0569^*^	−0.0722	−0.0425
				(0.0306)	(0.0452)	(0.0418)
Cognitive ability				0.2363^***^	0.2282^***^	0.2462^***^
				(0.0177)	(0.0262)	(0.0241)
Academic performance				0.0771^***^	0.0858^***^	0.0700^***^
				(0.0019)	(0.0031)	(0.0025)
Self-confidence				0.6490^***^	0.5832^***^	0.7204^***^
				(0.0400)	(0.0579)	(0.0564)
Health condition				0.1541^***^	0.1511^***^	0.1618^***^
				(0.0303)	(0.0433)	(0.0434)
Parental education				0.0979^***^	0.0927^***^	0.1020^***^
				(0.0053)	(0.0076)	(0.0073)
Family economic conditions				−0.0117	0.0269	−0.0466
				(0.0302)	(0.0458)	(0.0401)
One-child family				0.0676^**^	0.1048^**^	0.0431^*^
				(0.0295)	(0.0442)	(0.0197)
Nature of the school				−0.6516^***^	−0.6831^***^	−0.6232^***^
				(0.0496)	(0.0724)	(0.0682)
School quality				0.1695^***^	0.1896^***^	0.1549^***^
				(0.0216)	(0.0314)	(0.0300)
Regional characteristics				0.0761^***^	0.0886^***^	0.0654^***^
				(0.0163)	(0.0237)	(0.0227)
Pseudo *R*^2^	0.0038	0.0032	0.0055	0.0821	0.0823	0.0790
*N*	16,450			16,450	9,125	7,325

*** and **, respectively represent significant levels at 1 and 5% statistical levels.

According to the estimation results of gender-specific samples, the coefficients of extracurricular physical exercise in the female (Models 2 and 5) and male (Models 3 and 6) samples are positive (>0) and have passed the significance level test of 1% (* * *). This indicates that extracurricular physical exercise can significantly promote the self-education expectations of adolescents of both genders. After calculating the OR values of the independent variables in Models 5 and 6, the OR values corresponding to extracurricular physical exercise in the female and male samples were 1.1521 and 1.2657, respectively. This indicates that the likelihood of self-education expectations of adolescent female participating in extracurricular physical exercise increasing by one or more levels increases by an average of 15.21% compared to adolescent female who do not participate in extracurricular physical exercise, while the adolescent male proportion may reach 26.57%. This also means that extracurricular physical exercise has a greater effect on the self-education expectations of adolescent males than adolescent females under the same conditions.

According to control variables in Models 1–6, cognitive ability, academic performance, self-confidence, and health status in individual characteristics of adolescents have a positive impact on self-education expectations, regardless of whether they are female, male, or the entire sample, and have statistical significance. Adolescents with higher cognitive abilities, better academic performance, good health status, and higher confidence have higher self-education expectations. Age has a negative impact on adolescents’ self-education expectations. Extracurricular physical exercise has a lower improvement effect on self-education expectations in older adolescents than in younger ones. Regarding family characteristics, parental education, and only-child families significantly impact adolescents’ self-education expectations. The higher the education level of parents, the higher the self-education expectations of adolescents. Adolescents from only-child families have higher expectations for self-education than families with multiple children. The impact of family economic conditions on adolescents’ self-education expectations is insignificant. Regarding school characteristic variables, the nature of the school has a negative impact on the self-education expectations of adolescents; for example, the self-education expectations of adolescent students are higher in private schools than in public schools. The education quality positively affects the self-education expectations of young people; for example, the better the education quality, the higher the self-education expectations of young students. It presents that the self-education expectations of adolescents of different genders vary with age, registered residence, number of brothers and sisters, parental education, school nature, school running quality, and other conditions.

### Gender differences in the impact of extracurricular physical exercise on the self-education expectations of young people

Individual differences exist between adolescent students of different genders. The differences between individuals with different characteristics of the same gender should not be ignored (Yangyang and Rui, 2017). Adolescents’ family background is also important in participating in extracurricular physical exercise and self-education expectations (Pan et al., 2022). Furthermore, this study conducted group regression to examine the impact and heterogeneity of extracurricular physical exercise on self-education expectations of same-gender adolescent groups with different individual characteristics and family backgrounds. According to sample grouping by gender, we subdivided the samples based on the cognitive ability, academic performance, health status, confidence, parental education, and family economic conditions of students of the same gender to investigate the heterogeneity of the impact of extracurricular physical exercise on the self-education expectations of male and female groups with different characteristics. Based on the threshold of standardized score of cognitive ability, we divided the same- gender students into lower cognitive ability group, middle cognitive ability group and higher cognitive ability group. In the sub-group according to academic performance, the students of the same gender were considered to have poor academic performance on the basis of their average standardized scores in Chinese, mathematics and English, and those with scores of 65 or below were considered to have poor academic performance, those with a score greater than 65 and less than 75 were regarded as the intermediate academic performance group, and those with a score greater than 75 were regarded as the better academic performance group. According to the health status, students of the same gender who are healthy are divided into a group, and students of the same gender who are not healthy and unhealthy are divided into a group. According to the level of self-confidence, students of the same gender who were more confident and very confident were divided into a group, and students of the same gender who were less confident and less confident were divided into a group. In groups according to the educational level of the parents, parents with a maximum education duration of less than or equal to 9 years (junior secondary level and below) are assigned to the low education level group, those with a maximum education duration of more than 9 years and less than 15 years (junior secondary level and below) are assigned to the secondary education level group, and those with a maximum education duration of more than or equal to 15 years (junior secondary level and above) are assigned to the high education level group. In addition, according to the family economic condition, this study divides the adolescent students into the poor family, the middle-income family and the rich family. The group estimates are shown in Table 4^1^.

**Table 4:** The impact of extracurricular physical exercise on the educational expectations of adolescents of different genders.

Dependent variable Self education expectations		Female		Male		Full sample	
		Coefficient (SE)	OR value (SE)	Coefficient (SE)	OR value (SE)	Coefficient (SE)	OR value (SE)
Groups with different cognitive abilities	Low	0.1186(0.0700)	1.1259(0.0797)	0.2637^***^(0.0688)	1.3017^***^(0.0900)	0.1941^***^(0.0487)	1.2143^***^(0.0597)
	Medium	0.1932^***^(0.0602)	1.2131^***^(0.0724)	0.2764^***^(0.0622)	1.3184^***^(0.0807)	0.2356^***^(0.0428)	1.2657^***^(0.0538)
	High	0.0932(0.0743)	1.0977(0.0822)	0.1029(0.0801)	1.1083^***^(0.0859)	0.0922(0.0539)	1.0967(0.0588)
Different academic performance groups	poor	0.2817^**^(0.0894)	1.3255^**^(0.1195)	0.3085^***^(0.0649)	1.3608^***^(0.0878)	0.3037^***^(0.0521)	1.3549^***^(0.0708)
	Medium	0.1726^**^(0.0601)	1.1885^**^(0.0717)	0.2573^***^(0.0635)	1.2935^***^(0.0796)	0.2129^***^(0.0433)	1.2373^***^(0.0531)
	Preferably	0.0620(0.0623)	1.0640(0.0656)	0.1442(0.0961)	1.1551(0.0995)	0.0890(0.0501)	1.0931(0.5542)
Groups with different health conditions	Unhealthy groups	0.1738^**^(0.0691)	1.1898^***^(0.0829)	0.2427^***^(0.0502)	1.3779^***^(0.1026)	0.3206^***^(0.0740)	1.2747^***^(0.0647)
	Healthy group	0.1267^***^(0.0469)	1.1350^***^(0.0532)	0.1607^***^(0.0328)	1.2222^***^(0.0565)	0.2007^***^(0.0469)	1.1743^***^(0.0385)
Different groups with different levels of confidence	Lack of confidence	0.0999(0.0957)	1.1051(0.1079)	0.2981(0.1963)	1.3473(0.9313)	0.2000(0.1673)	1.2213(0.8838)
	Relatively confident	0.1466^***^(0.0423)	1.1579^***^(0.0490)	0.2224^***^(0.0434)	1.2491^***^(0.0536)	0.1820^***^(0.0300)	1.1997^***^(0.0360)
Different groups of parents with different levels of education	≤9 years	0.1564^***^(0.0520)	1.1693^***^(0.0606)	0.2775^***^(0.0524)	1.3198^***^(0.0687)	0.2163^***^(0.0365)	1.2415^***^(0.0453)
	9 < & < 15	0.2060^***^(0.0765)	1.2287^***^(0.0933)	0.2382^***^(0.0754)	1.2689^***^(0.0949)	0.2123^***^(0.0534)	1.2366^***^(0.0655)
	≥15 years	0.0005(0.0932)	1.0005(0.0947)	0.0654(0.1023)	1.0676(0.5084)	0.0311(0.0681)	1.0317(0.0709)
Different groups with different family economic conditions	Poverty	−0.0138(0.1111)	0.9863(0.1101)	0.1345(0.1063)	1.1439(0.1226)	0.0606(0.0760)	1.0625(0.0816)
	Medium	0.1780^***^(0.0440)	1.1971^***^(0.0526)	0.2622^***^(0.0455)	1.2997^***^(0.0588)	0.2206^***^(0.0313)	1.2468^***^(0.0391)
	Affluent	−0.0286(0.1259)	0.9718(0.1245)	0.1395(0.1238)	1.1497(0.1367)	0.0709(0.0879)	1.0734(0.0931)

*** and **, respectively represent significant levels at 1 and 5% statistical levels.

From Table 4, it can be seen that the degree of influence of extracurricular physical exercise on the self-education expectation of adolescent groups with different characteristics of the same gender also shows certain differences, which are analyzed as follows.

First of all, from the perspective of cognitive ability difference groups, the coefficients of the influence of extracurricular physical exercise on the self-education expectation of the group of female with low and medium cognitive ability are 0.1186 and 0.1932 respectively, and the coefficients correspond to OR values of 1.1259 and 1.2131 respectively, and the group of female with medium cognitive ability have passed the significance level test of 1%. This indicates that extracurricular physical activity can significantly enhance the self-educational expectations of female adolescent students with low and medium cognitive ability levels, and the enhancement effect on the self-educational expectations of female students with higher cognitive ability groups is not significant. Compared with the group of female students with low cognitive ability who do not participate in extracurricular sports. The likelihood of increasing their self-educational expectations by one or more tiers will increase by an average of 21.31%. This indicates that, all other things being equal, extracurricular physical activity has a greater effect on the self-educational expectations of the group of female with medium cognitive abilities than the group of female with low cognitive abilities. It is worth noting that the coefficient of the effect of extracurricular physical exercise on the self-teaching expectations of the group of female with high cognitive ability is 0.0932, but it does not pass the significance level test. It indicates that the effect of extracurricular physical exercise in promoting self-educational expectations of the group of female with high cognitive ability is not significant. From the sample of male, the coefficient of influence of extracurricular physical exercise on the self-educational expectations of male’ groups with low and medium cognitive ability is 0.2637 and 0.2764 respectively, and the corresponding OR values of the coefficients are 1.3017 and 1.3184, and both of them have passed the significance level test of 1%. It shows that extracurricular physical exercise can significantly enhance the self-educational expectations of male students with low and medium cognitive ability levels. Compared with the group of male with low cognitive ability who do not participate in extracurricular physical activity, the group of male with low cognitive ability who participate in extracurricular physical activity will increase the likelihood of increasing their self-educational expectations by one or more levels by an average of 30.17%; and compared with the group of male with medium cognitive ability who do not participate in extracurricular physical activity, the group of male with medium cognitive ability who participate in extracurricular physical activity will increase the likelihood of increasing their self-educational expectations by the likelihood of one or more tiers will increase by an average of 31.84%. It should also be noted that the coefficient of the effect of extracurricular physical education on the self-educational expectations of the group of male with higher cognitive abilities is not statistically significant, indicating that the effect of extracurricular physical education on the self-educational expectations of the group of male with higher cognitive abilities is not significant.

Second, regarding different academic performance groups, the impact coefficients of extracurricular physical exercise on self-education expectations of female students with poor and medium academic performance are 0.2817 and 0.1726, respectively. The corresponding OR values of the coefficients are 1.3255 and 1.1885, respectively. Both passed the significance level test of 5% (* *), but the impact coefficients on self-education expectations of female students with good academic performance did not pass the significance level test. It indicates that extracurricular physical exercise can significantly enhance the self-education expectations of female students with poor or moderate academic performance while not affecting female students with good academic performance is insignificant. Compared to female with poor academic performance who do not participate in extracurricular physical exercise, the probability of female with poor academic performance participating in extracurricular physical exercise increasing their self-education expectations by one or more levels may increase by an average of 32.55%. This increase is 18.15% for female with moderate academic performance. It indicates that the effect of extracurricular physical exercise on improving self-teaching expectations of female students with poor academic performance is slightly greater than that of female students with moderate academic performance under the same conditions. The improvement effect of extracurricular physical exercise on the self-education expectations of female students with better academic performance is insignificant. The impact of extracurricular physical exercise on self-education expectations of male students with poor, medium, and better academic performance is similar to that of female students in the male sample. The self-education expectations of male students with poor and moderate academic performance can be significantly improved, but the improvement effect on self-education expectations of male students with higher academic performance is insignificant. Compared to male with poor academic performance who do not participate in extracurricular physical exercise, the self-education expectations of male with poor academic performance who participate in extracurricular physical exercise increase by an average of 36.08%, with an increase of 29.35% for male with moderate academic performance.

Third, from the perspective of the health status and self-confidence difference groups, the coefficients of the effect of extracurricular physical activity on the self-educational expectations of the groups of male and female with different health status are positive and significant, respectively. From the perspective of the less healthy group, relative to the group of female with poor health who do not participate in extracurricular physical activity, the group of female with poor health who participate in extracurricular physical activity will increase the likelihood of increasing their self-educational expectations by one or more tiers by an average of 18.98%, and this increase in the group of male reaches 37.79%, which is much larger than that of female. In terms of the healthy group, healthy female who participate in extracurricular physical activity are on average 13.50% more likely to increase their self-education expectations by one or more levels than healthy female who do not participate in extracurricular physical activity, whereas the increase in the male’ group is 22.22%, which is still greater than that of the female. The likelihood of increasing self-education expectations by one or more levels increases by an average of 18.98%, with the male population increasing by 37.79%, far more than the female population. Regarding health, the likelihood of increasing self-education expectations by one or more levels increases by an average of 13.50%, while the increase in the male population is 22.22%, which is still higher than that of the female population. Regarding confidence differences, extracurricular physical exercise has an insignificant impact on the self-education expectations of female and male who lack confidence. Extracurricular physical exercise significantly positively impacts the self-education expectations of more confident female and male groups. Participation in extracurricular physical exercise increases the likelihood of more confident female and male students’ self-education expectations by one or more levels by 15.79 and 24.91%, respectively. Different confidence levels are also important factors that influence adolescents’ self-education expectations through extracurricular physical exercise.

Fourth, from the perspective of family background, parents’ education level and family economic conditions show obvious group differences. Extracurricular physical exercise has a significant positive impact on the self-education expectations of female and male parents with low (≤9 years) and moderate (9< and <15 years) education levels. Participation in extracurricular physical exercise increases the likelihood of self-education expectations of female and male parents with lower education levels by one or more levels by 16.93 and 31.98%, respectively. It also increases the likelihood of self-education expectations of female and male parents with medium education levels by one or more levels by 22.87 and 26.89%, respectively. Extracurricular physical exercise has no significant impact on the self-education expectations of female and male parents with higher levels of education (≥15 years).

Regarding differences in family economic conditions, extracurricular physical exercise has no significant impact on the self-education expectations of female and male from impoverished and affluent families. However, it positively and significantly impacts the self-education expectations of female and male from middle-income families. Participation in extracurricular physical exercise increases the likelihood of female and male from middle-income families increasing their self-education expectations by one or more levels by 19.71 and 29.97%, respectively.

### The mechanism of extracurricular physical exercise affecting the self-education expectations of adolescents

The above analysis indicates that extracurricular physical exercise can enhance the self-education expectations of adolescents to varying degrees. The impact on the self-education expectations of different groups of adolescents with different characteristics varies significantly. So, how does extracurricular physical exercise impact teenagers’ self-education expectations? To clarify this issue, the following section further analyzes how extracurricular physical exercise affects adolescents’ self-education expectations. Existing research has found that improving cognitive ability, academic performance, health level, and self-confidence are important ways for adolescents to enhance their self-education expectations through extracurricular physical exercise (Zarazaga-Peláez et al., 2024). The “Executive Function Hypothesis” suggests that physical exercise primarily promotes individual cognitive and executive function development via three subcomponents: inhibitory control, cognitive flexibility, and working memory. Individuals with good cognitive and executive abilities frequently have stronger psychological resilience, including self-esteem, self-confidence, self-control, perseverance, higher academic achievement, and goal orientation (Liu, 2018; Jiang et al., 2018). The self-efficacy theory proposes a social psychological mechanism by which physical exercise affects adolescents’ self-education expectations (Bandura, 2004; Mesquita et al., 2012). The correlation between sports activities and self-efficacy as the central mechanism of behavioral reasons is the strongest and most stable (Lian-Cheng et al., 2015). Exercise provides positive opportunities for individuals, especially those frequently encountering setbacks, to rebuild their self-concept, mindfulness level, and self-efficacy. It has a significant positive impact (Pu et al., 2017; Wei, 2018; Guo and Wei, 2020). It helps individuals maintain good mental health under continuous pressure, actively reassess and stimulate their potential, thereby establishing stronger confidence, a more positive mindset, and a higher pursuit of goals (Chen et al., 2018). Based on this, this study will use four variables: health status, cognitive ability, academic performance, and confidence as the mechanism variables to empirically test whether extracurricular physical exercise can have a positive impact on self-education expectations by promoting adolescent health level, cognitive ability, academic performance, and self-confidence. According to the basic principle of mechanism testing, the mechanism variable is replaced with the dependent variable of the ordered logistic benchmark regression model (Table 3). If the mechanism is established, the independent variable should meet expectations and have statistical significance. Table 3 indicates that the four variables of health status, cognitive ability, academic performance, and confidence have a significant positive impact on the self-education expectations of adolescents, which is beneficial for significantly improving their self-education expectations. According to the mechanism testing approach, the next step is to examine whether extracurricular physical exercise significantly impacts the abovementioned four variables. Therefore, ordered logistic regression model was conducted using health level, cognitive ability, academic performance, and confidence as dependent variables and self-education expectations as independent variables. Table 5 illustrates the estimated results of the mechanism analysis model.

**Table 5:** Results of mechanism analysis.

Independent variable: extracurricular physical exercise	Dependent variable: health status			Dependent variable: cognitive ability		
	(1)	(2)	(3)	(4)	(5)	(6)
	Female	Male	Full sample	Female	Male	Full sample
Extracurricular physical exercise	0.3284^***^	0.2454^***^	0.4180^***^	0.2459^***^	0.1897^***^	0.2157^***^
	(0.0348)	(0.0480)	(0.0505)	(0.0415)	(0.0421)	(0.0295)
Other variables	Control	Control	Control	Control	Control	Control
*N*	9,125	7,325	16,450	9,125	7,325	16,450

Independent variable: extracurricular physical exercise	Dependent variable: academic performance			Dependent variable: confidence		
	(1)	(2)	(3)	(4)	(5)	(6)
	Female	Male	Full sample	Female	Male	Full sample
Extracurricular physical exercise	0.0982^**^	0.2147^***^	0.1580^***^	0.4329^***^	0.4490^***^	0.4121^***^
	(0.0417)	(0.0416)	(0.0294)	(0.0440)	(0.0621)	(0.0627)
Other variables	Control	Control	Control	Control	Control	Control
*N*	9,125	7,325	16,450	9,125	7,325	16,450

*** and **, respectively represent significant levels at 1 and 5% statistical levels.

Table 5 displays that extracurricular physical exercise positively impacts the health level, cognitive ability, academic performance, and self-confidence of female students, male students, and the entire sample of adolescents, and all have passed the 1% significance level test. It indicates that extracurricular physical exercise significantly improves adolescents’ health, cognitive ability, academic performance, and confidence. They can also significantly enhance their self-education expectations due to their health, cognitive ability, academic performance, and self-confidence. This indicates that extracurricular physical exercise can positively affect teenagers’ self-education expectations by improving their health status, cognitive ability, academic performance, and confidence. They verified that health level, cognitive ability, academic performance, and self-confidence are important channels for physical exercise to enhance teenagers’ self-education expectations.

## Discussion

This study explores the impact and mechanism of extracurricular physical exercise on the self-education expectations of adolescent female and male, as well as the heterogeneity of the impact of extracurricular physical exercise on self-education expectations of different types of female and male, against the backdrop of vigorously advocating to reduce the academic burden of adolescents and increasing physical exercise. It is of great significance to clarify the channels and mechanisms by which extracurricular physical exercise promotes the self-education expectations of young people, as well as to recognize the importance of extracurricular physical exercise for the ultimate academic achievement of young people.

This study discovered that extracurricular physical exercise significantly positively impacts teenagers’ self-education expectations, which can significantly improve teenagers’ self-education expectations. Moreover, the overall improvement effect on male’ self-education expectations is greater than that of female. Grouping regression analysis for different types of same- gender groups revealed varying degrees of differences in the improvement effect of extracurricular physical exercise on self-education expectations among groups with different cognitive abilities, academic performance, health status, self-confidence, parents’ education levels, and family economic conditions.

Previous studies have focused on the impact of classroom physical exercise on adolescent male’ and female’ self-education expectations and academic achievements. However, few studies considered the impact of extracurricular physical exercise on the self-education expectations of adolescent male and female. This study discovered the heterogeneity of the impact of extracurricular physical exercise, with weekends as a typical time point, on the self-education expectations of different same- gender groups. This discovery is more in-depth and important than other literature in this study.

First, regarding the characteristics of the group itself, the effect of extracurricular physical exercise on the self-education expectations of female with moderate cognitive abilities is greater than that of female with low cognitive abilities, whereas the effect on the self-education expectations of male with moderate and low cognitive abilities is relatively similar. The differences in cognitive abilities also lead to varying degrees of impact of extracurricular physical exercise on the self-education expectations of adolescents. The improvement effect of extracurricular physical exercise on the self-education expectations of female and male with poor academic performance is greater than its effect on female and male with moderate academic performance. The improvement effect on the self-education expectations of female and male with higher cognitive ability or better academic performance is insignificant.

The possible reason is that adolescent students with high cognitive abilities or good academic performance have relatively high self-education expectations, and the marginal utility of extracurricular physical exercise to enhance the self-education expectations of such groups is insignificant. Participation in extracurricular physical exercise may significantly promote the self-education expectations of more confident female and male groups, but the promotion effect on the group lacking confidence is insignificant. Students who lack confidence frequently have weak psychological resilience, and internal psychological and personality factors dominate and constrain them.

Second, regarding family characteristics, this study discovered that the class differences represented by parents’ education level and family economic conditions are important factors that affect adolescent students’ extracurricular physical exercise and self-education expectations. Extracurricular physical exercise has a smaller effect on the self-education expectations of female with lower parental education than female with moderate parental education, while male have the opposite effect. The impact of extracurricular physical exercise on the self-education expectations of female and male parents with higher education levels is insignificant. The possible reason is that the higher the education level of parents, the higher their self-education expectations of their children are influenced by their words, deeds, and experiences (as verified in the descriptive statistical analysis above). The effect of external factors, such as extracurricular physical exercise, on the self-education expectations of these groups is insignificant. This indicates the positive role of extracurricular physical exercise in narrowing the educational expectations gap between children from families with higher parental education levels and children with moderate or lower parental education levels. This study also discovered that extracurricular physical exercise promotes the self-education expectations of female and male from middle-income families, but the effect on the self-education expectations of female and male from impoverished and affluent families is insignificant. Some studies have indicated a positive correlation between the education level of workers and their income (Chen et al., 2018). Therefore, the middle-income group is the most anxious in education in China. Based on the general psychology of “not letting children lose at the starting line, “this group also arranges various subject training for children on weekends, seriously squeezing their sports time. Based on this background, this study’s conclusions provide positive evidence for increasing extracurricular physical activity among adolescent students and alleviating educational anxiety in middle-income families.

This study also discovered that extracurricular physical exercise can positively affect teenagers’ self-education expectations by promoting improved health status, cognitive ability, academic performance, and self-confidence. This study clarified the mechanisms and channels through which extracurricular physical exercise improves self-education expectations among adolescents. This study is more unique than existing studies. The insights derived from this study have profound implications for educational policies and family education strategies. They advocate for the integration of physical activities into educational curriculums, emphasizing their efficacy in enhancing adolescents’ educational expectations and achievements. Educational policies should, therefore, encourage varied participation in physical activities, paying special attention to students with different cognitive and academic profiles. Additionally, the role of family background in influencing the outcomes of physical exercise on educational expectations cannot be understated, especially under the “double reduction” policy aimed at alleviating academic stress. Enhancing physical education infrastructure in schools, promoting innovative exercise methods, and providing a wide range of extracurricular activities are essential steps in this direction. Such comprehensive approaches can significantly boost adolescents’ health, cognitive capabilities, and confidence, ultimately fostering higher educational aspirations and achievements.

Several limitations of this study must also be considered. First, cross-sectional data may have certain limitations in causal inference because this study only used cross-sectional data from CEPS 2013–2014. Teenagers’ extracurricular physical exercise and self-education expectations may be a gradual process of adjustment and change, and cross-sectional data cannot fully reveal their changing trajectory. Future research can use longitudinal panel data to describe the trajectory of changes in extracurricular physical exercise and self-education expectations among different adolescents, revealing their internal evolution mechanisms. Second, this study examined the effect of extracurricular physical exercise on the self-education expectations of different adolescent groups, typically at weekends. It did not comprehensively examine the impact and role of other extracurricular periods, such as evening and non-class hours, on the self-education expectations of adolescents. If all extracurricular periods of physical exercise were examined, the results may be more convincing. Finally, self-education expectations are explored from the perspective of academic qualifications, that is, the highest level of educational attainment set by students themselves. This single dimensional measurement method appears somewhat one-sided when delving into the complex concept of educational expectations. Future research needs to introduce more diverse perspectives and analytical dimensions.

## Conclusion

This investigation into the impact of extracurricular physical exercise on adolescents’ self-education expectations reveals a significant gender disparity, with male showing a more pronounced enhancement than female. The influence of physical exercise is also modulated by individual factors such as cognitive abilities, academic performance, health status, and self-confidence, as well as familial influences like parents’ education level and economic conditions. Notably, female students with moderate cognitive abilities and lower academic performance, along with male students facing challenges in academic performance and health, show the most substantial improvements in self-education expectations. These findings highlight the critical role of extracurricular physical exercise in fostering academic aspirations and underscore the necessity to tailor these activities to the diverse needs of different adolescent groups.

## Data Availability

The raw data supporting the conclusions of this article will be made available by the authors, without undue reservation.

## Footnotes

^1^Limited to space, only the estimated coefficients of independent variables and OR value, other control variables can be obtained from the author if necessary, the same as below.
